# Fangchinoline inhibits growth and biofilm of *Candida albicans* by inducing ROS overproduction

**DOI:** 10.1111/jcmm.18354

**Published:** 2024-04-30

**Authors:** Longfei Yang, Xiaonan Wang, Zhiming Ma, Yujie Sui, Xin Liu

**Affiliations:** ^1^ Jilin Provincial Key Laboratory on Molecular and Chemical Genetics The Second Hospital of Jilin University Changchun China; ^2^ Department of Orthopedics The Second Hospital of Jilin University Changchun China; ^3^ Department of Gastrointestinal Nutrition and Hernia Surgery The Second Hospital of Jilin University Changchun China; ^4^ Eye Center, The Second Hospital of Jilin University Changchun China

**Keywords:** antifungal, biofilm, *Candida albicans*, fangchinoline, ROS, virulence factor

## Abstract

Infections caused by *Candida* species, especially *Candida albicans*, threaten the public health and create economic burden. Shortage of antifungals and emergence of drug resistance call for new antifungal therapies while natural products were attractive sources for developing new drugs. In our study, fangchinoline, a bis‐benzylisoquinoline alkaloid from Chinese herb *Stephania tetrandra* S. Moore, exerted antifungal effects on planktonic growth of several *Candida* species including *C. albicans*, with MIC no more than 50 μg/mL. In addition, results from microscopic, MTT and XTT reduction assays showed that fangchinoline had inhibitory activities against the multiple virulence factors of *C. albicans*, such as adhesion, hyphal growth and biofilm formation. Furthermore, this compound could also suppress the metabolic activity of preformed *C. albicans* biofilms. PI staining, followed by confocal laser scanning microscope (CLSM) analysis showed that fangchinoline can elevate permeability of cell membrane. DCFH‐DA staining suggested its anti‐*Candida* mechanism also involved overproduction of intracellular ROS, which was further confirmed by N‐acetyl‐cysteine rescue tests. Moreover, fangchinoline showed synergy with three antifungal drugs (amphotericin B, fluconazole and caspofungin), further indicating its potential use in treating *C. albicans* infections. Therefore, these results indicated that fangchinoline could be a potential candidate for developing anti‐*Candida* therapies.

## 1 INTRODUCTION

Diseases caused by fungal pathogens affect millions of people worldwide and annual death resulting from fungal infections approaches 2 million and is still rising.^1^ The reasons for the this rise in fungal infections include the increase in the susceptible population (due to HIV infections, immunosuppression therapy and diabetes mellitus), late diagnosis, the prevalence of drug resistance, and most importantly, the scarcity of antifungal drugs.^2^ Among these pathogens, *Candida* species, especially *Candida albicans*, are the major causes of fungaemia.^3^, ^4^, ^5^ Besides Candidaemia, *C. albicans* can also cause mucosal infections such as oral thrush and vulvovaginal candidiasis, although this fungus belongs to one of the commensal fungi residing in gastrointestinal and vaginal tracts.^2^ The pathogenicity of *C. albicans* is closely associated with its ability to form hypha, which enables its difficulty to be engulfed by and easy escape from phagocytes, even death of phagocytes.^6^ *C. albicans* hyphae also express superoxide dismutase 5 (SOD5) that detoxifies reactive oxygen species (ROS), and secret pore‐forming candidalysin that damages tissues.^6^ In addition, hyphae, along with pseudohyphae and yeasts, are essential components of *C. albicans* biofilms that usually formed on surfaces of mucosae and medical devices.^2^, ^7^ The extracellular exopolysaccharide (EPS) matrix encasing cells within biofilms exerts protection against antifungal drugs and immune attacks from host, increasing the tolerance of biofilms to antifungal therapeutics.^8^, ^9^ The resistance of biofilms to antifungal drugs often necessitates replacement of medical devices, exerting additional economic burden on patients.^2^ This, along with above‐mentioned reasons for rise in incidence, as well as the emergence of new drug‐resistant fungal pathogens, highlights the necessity to develop novel antifungal therapies.^7^, ^10^

**FIGURE 1:**
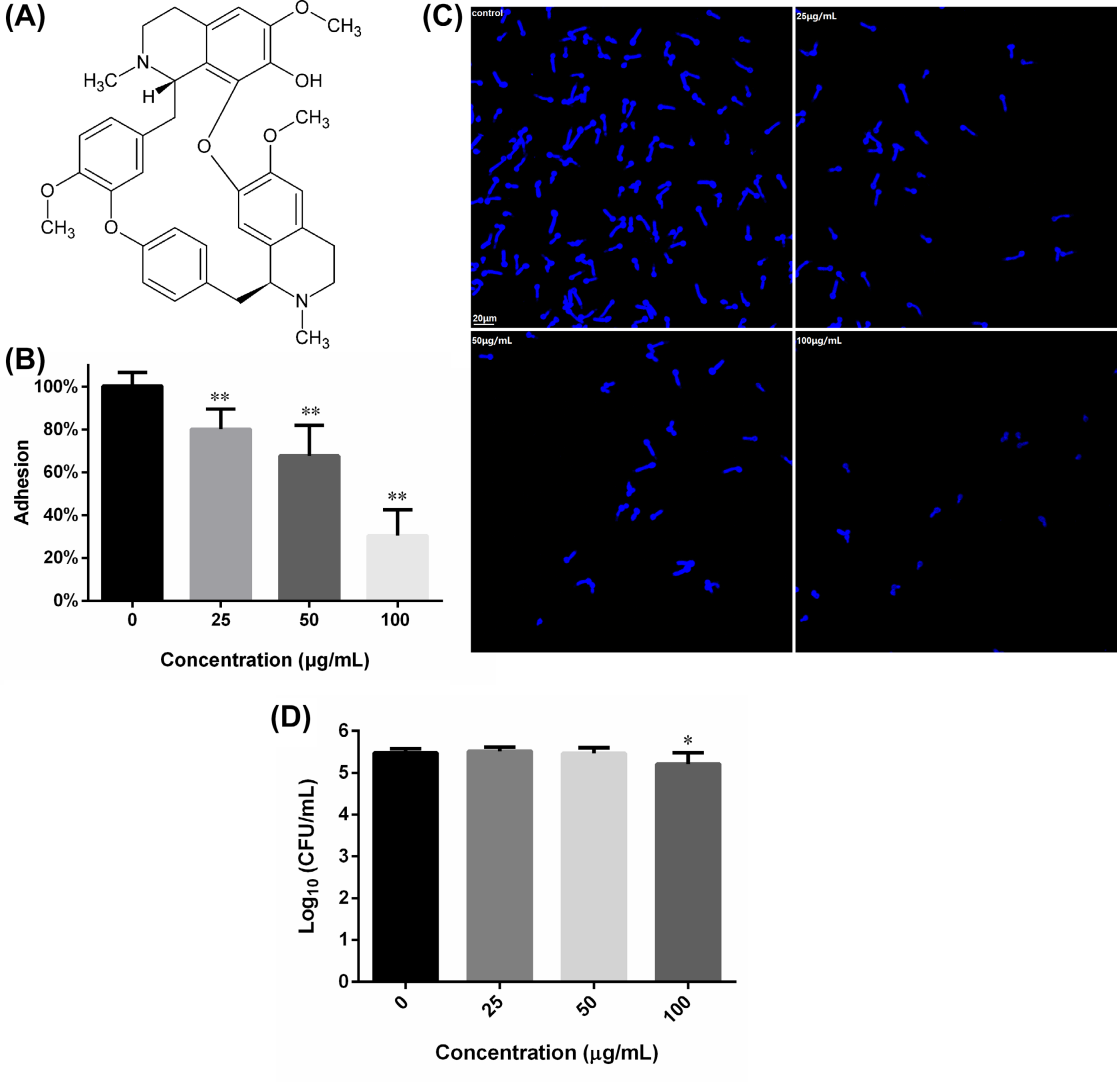
The chemical structure of FN and the suppression of FN on the adhesion of *Candida albicans* cells to polystyrene surfaces. (A) The chemical structure of FN. (B) FN inhibited the adhesion of *C. albicans* SC5314 to polystyrene surfaces. Adhesion was considered as the relative viability of adherent fungal cells determined by MTT assay. (C) Representative graphs of adherent *C. albicans* cells left by FN treatment and PBS washing. (D) The total viable cells in each group after 1.5 h treatment with different concentrations of FN at 37°C (before PBS washing). *, *p* < 0.05 and **, *p* < 0.01.

In the past decades, there are continuous efforts to search and develop antifungal agents, from natural products, especially from herbal plants.^2^, ^11^ Among Chinese traditional medicinal plants, *Stephania tetrandra* S. Moore (fang ji in Chinese) is used to cure multiple diseases and harbours various kinds of bioactive compounds including mainly alkaloids, as well as flavonoids and steroids.^12^ Fangchinoline (FN, Figure 1A), is one such alkaloids containing bis‐benzylisoquinoline structure. FN has demonstrated antitumour activities in various kinds of cancer cells,^13^ antiviral activities against porcine epidemic diarrhoea virus, human coronavirus strain OC43, SARS‐CoV‐2 and human immunodeficiency virus^14^, ^15^, ^16^, ^17^ and anti‐inflammatory activities in rat model of rheumatoid arthritis.^18^ FN can also ameliorate diabetes‐related nephropathy^19^ and retinopathy induced by streptozotocin.^20^ Although FN has been reported to own weak or no antifungal activity against plant fungal pathogens such as *Ascosphaera apis*,^21^ *Phomopsis adianticola*, *Altermaria adianticola*, *Colletotrichum fructicola*, *Pestalotiopsis theae*, *Phoma adianticola* and *Gibberella zeae*,^22^ with all minimal inhibitory concentration (MIC) above 200 μg/mL, its effects on human fungal pathogens, such as *C. albicans* have never been evaluated. In this study, we investigated the effects of FN on the planktonic growth and virulence factors of *C. albicans*, and its underlying mechanisms.

## 2 MATERIALS AND METHODS

### 2.1 Strains, culture conditions and chemicals

*Candida albicans* SC5314 and *C. albicans* ATCC10231, as well as four other *Candida* species (Table 1), were obtained from CGMCC (China General Microbiological Culture Collection Center) and kept on YPD agar at 4°C in fridge. Prior to each assay, one colony was propagated in YPD medium for 18 h at 28°C with a rotation of 140 rpm.

**TABLE 1:** The antifungal activities of FN against *Candida* species.

Fungal strains	FN		Amphotericin B	
	MIC (μg/mL)	MFC (μg/mL)	MIC (μg/mL)	MFC (μg/mL)
*C. albicans* SC5314	50	50	0.625	1.25
*C. albicans* ATCC 10231	50	200	0.3125	1.25
*C. glabrata* ATCC 2001	50	200	0.625	2.5
*C. parapsilosis* ATCC 22019	25	100	0.625	2.5
*C. krusei* ATCC 6258	12.5	50	1.25	2.5
*C. tropicalis* ATCC 7349	50	100	0.3125	2.5

Fangchinoline (Purity > 98%, HPLC), DCFH‐DA‐containing ROS kit, dimethyl sulfoxide (DMSO) and N‐acetyl‐cysteine (NAC) were purchased from Solarbio (Beijing, China). MTT, XTT, fluconazole and Amphotericin B (AmB) were purchased from Sangon (Shanghai, China). PI and Calcofluor White (CFW), were purchased from Sigma (Shanghai, China). Caspofungin acetate was purchased from Aladdin Scientific (Shanghai, China). Syto 9 and RPMI‐1640 medium (powder) were purchased from Thermo (Shanghai, China). FN was dissolved in DMSO at a concentration of 10 mg/mL as stocking solution. AmB, prepared in DMSO at 1 mg/mL, was used as the positive control.

### 2.2 Antifungal activity test

Broth microdilution assay following CLSI‐M27‐A3 guidelines was employed to test the antifungal activity of FN against the above‐mentioned *Candida* strains. Fungal cells (2 × 10^3^ cells/mL in RPMI‐1640 medium) were exposed to a serial of concentrations of FN for 24 h at 37°C to monitor the fungal growth in 96‐well plates. The MIC was set as the lowest concentration at which in the corresponding wells no fungal growth was observed. When fungal cultures in MIC tests exposed to FN (above MIC) were transferred to YPD agar to allow growth for 24 h, the lowest concentration where no fungal colony was found was defined as minimal fungicidal concentration (MFC).^23^

### 2.3 Adhesion to polystyrene surfaces

Hundred microlitres of *C. albicans* SC5314 suspension (10^6^ cells/mL in RPMI‐1640 medium) was filled into each well of 96‐well plate and treated with 0, 25, 50 and 100 μg/mL FN for 90 min at 37°C. After washing with PBS, 100 μL fresh medium and 10 μL MTT solution were added, followed by a 3‐h incubation in dark. Later, the supernatant of each well was decanted and DMSO was added to dissolve formazan. The optical density at 490 nm of each well was read by microplate reader (VarioSkan, Thermo) to calculate the relative adhesion.^7^

CFW staining was also performed to show directly the influence of FN on adhesion to 96‐well plate bottom. After treatment with FN for 90 min and washing, the fungal cells left on surfaces were stained with CFW and photographed. To explore whether the effects of FN on adhesion were due to the killing activity of FN, the fungal cultures in each group above‐mentioned before washing were taken out, serially diluted and plated on SD agar. After 24‐h incubation at 37°C, the colony forming units (CFU) on each agar plate were counted to calculate the number of viable cells in each group.

### 2.4 Time‐kill assay

This assay was performed in RPMI‐1640 medium at 28°C with a rotation of 140 rpm. *C. albicans* cells (10^4^, 10^5^ and 10^6^ cells/mL) were exposed to 12.5, 25, 50, 100 and 200 μg/mL of FN. At the 2nd, 4th, 6th, 8th, 12th and 24th hour after FN exposure, 100 μL of the suspension was retrieved from each group, serially diluted and spread on SD agar. The CFU on each agar were counted after 24 h incubation at 37°C.

### 2.5 Hyphal growth

The *C. albicans* cells from 18‐h grown cultures were diluted to get a density of 10^6^ cells/mL in RPMI‐1640 medium, which was further treated with 0, 25, 50 and 100 μg/mL FN at 37°C for 4 h. Cellular morphologies were photographed by a microscope (Olympus IX81, Japan)‐linked camera.

### 2.6 Biofilm test

The 96‐well plates‐based biofilm formation of *C. albicans* SC5314 was used as employed before.^24^ Biofilms formed in the presence of 0, 12.5 25, 50, 100 and 200 μg/mL FN for 24 h at 37°C were washed with PBS, prior to the XTT reduction assay. To evaluate the influence of FN on biofilm development, biofilms produced in the absence of any drug were washed with PBS and further incubated with fresh medium containing various concentrations of FN for another 24 h. Then, XTT assay was performed to determine the biofilm viability.

To get a view of biofilm structures, confocal laser scanning microscope (CLSM, Olympus FV1000, Japan) was employed to record the three‐dimensional structures of *C. albicans* SC5314 biofilms formed in the presence of 0, 25, 50 and 100 μg/mL FN. Biofilms were stained with 10 μM Syto9 and washed with PBS. 40× objective and a step‐size of 2 μm were used to record the biofilms in 3D mode. Recordings were re‐built with Imaris 7.2.3 (Bitplane AG, Zurich, Switzerland) as described previously.^25^

### 2.7 EPS production

The 24‐h matured biofilms in 24‐well plates were treated with 0, 25, 50 and 100 μg/mL FN for 24 h at 37°C, followed by washing with PBS. A total quantity of 0.9% NaCl solution, 5% phenol and 0.2% hydrazine sulphate were added into wells in a ratio of 1:1:10 and kept in dark for 60 min. Microplate reader was used to determine the optical density of each well at 490 nm.^23^

### 2.8 Membrane permeability test

Cell membrane‐impermeable fluorescent dye PI was used to assay the permeability of *C. albicans* cell membrane. Fungal cells challenged with 0, 25, 50 and 100 μg/mL FN for 4 h at 37°C were stained with10 μM PI for 10 min. After washing with PBS, the fluorescence of *C. albicans* cells was examined using CLSM.

### 2.9 ROS determination

The widely used ROS‐detecting fluorescent dye DCFH‐DA (final concentration: 10 μM) was employed to stain the *C. albicans* cells that were treated with 0, 25, 50 and 100 μg/mL FN for 4 h. Cells with excessive intracellular ROS production emit green fluorescence, which could be detected and recorded by CLSM.

### 2.10 NAC rescue test

To further confirm the role of ROS in the antifungal activity of FN, 150 μg/mL NAC was supplemented in RPMI‐1640 medium. Biofilms formed in the absence and presence of 100 μg/mL FN, in the medium containing NAC or not, were subjected to XTT assay to determine the viability. Biofilms of these groups were also photographed using inverted microscope.^26^ In addition, the influence of 150 μg/mL NAC on the MIC of FN against *C. albicans* SC5314 was also evaluated, as following the protocols described in part 2.2.

### 2.11 Checkerboard assay

The MIC‐based checkerboard assay was performed to test the interactions between FN and other antifungal drugs (amphotericin B, fluconazole and caspofungin), as described previously.^26^

### 2.12 Statistical analysis

Data shown were mean ± SD from triplicates in three independent tests and GraphPad Prism 6.02 was used to perform Student *t* test to obtain the statistical significance.

## 3 RESULTS

### 3.1 Antifungal susceptibility

As shown in Table 1, fangchinoline inhibited all the six strains tested, with MIC ranging from 12.5 μg/mL against *C. krusei* to 50 μg/mL against *C. albicans*, *C. tropicalis* and *C. glabrata*. The MFC of fangchinoline also varied in a similar trend roughly. Being able to form biofilms easily, *C. albicans* SC5314 was selected for further analysis.

### 3.2 Adhesion assay

As shown in Figure 1B, FN could impede the adhesion of *C. albicans* to the abiotic polystyrene surfaces of 96‐well plates, in a concentration‐dependent way. A total quantity of 25, 50 and 100 μg/mL FN could inhibit the adhesion by about 20%, 32% and 70%, as compared to the FN‐free control. This inhibition could also be confirmed by microscopic graphs of adherent fungal cells that experienced FN treatment and PBS washing, which showed that increasing FN concentration caused less cells left on polystyrene surfaces (Figure 1C). To validate whether the inhibition of adhesion was due to the decrease in viable cell number caused by FN treatment, the CFU in each group were counted. As shown in Figure 1D, 25 and 50 μg/mL FN did not reduce the CFU number significantly, suggesting that FN, at these concentrations, did inhibit adhesion without influencing the cell viability. However, 100 μg/mL FN reduce the CFU number significantly, as compared to the control. This, combined with results of 25 and 50 μg/mL FN, suggested that the killing effect may further contribute to the inhibition of adhesion.

### 3.3 Time‐kill kinetics

**FIGURE 2:**
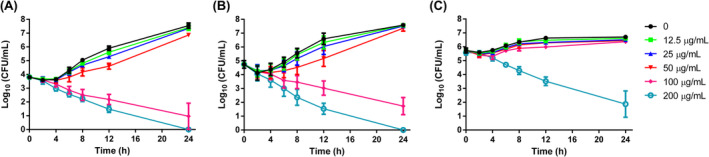
The time‐kill curves of FN in *Candida albicans* SC5314 cells. *Candida* cells with different initial inoculum densities (10^4^ cells/mL (A), 10^5^ cells/mL (B) and 10^6^ cells/mL (C)) were treated with 0, 12.5, 25, 50, 100 and 200 μg/mL FN for 24 h. At indicated time points, 100 μL aliquot was taken from each group, diluted and spread on SD agar to count the viable cell number of each group.

The fungicidal effects of FN on *C. albicans* cells were dependent on both the FN concentration and the initial fungal cell density, as shown in Figure 2. *Candida* cells exposed to sub‐MIC 12.5 and 25 μg/mL FN showed similar curves to drug‐free control, as expected, in all the three assays with different initial cell density. While 50 μg/mL only retard the fungal growth to some extent in assays with initial density of 10^4^ and 10^5^ cells/mL (Figure 2A,B), this concentration was MFC in assays with initial density of 2 × 10^3^ cells/mL (Table 1). When the cell density increased to 10^6^ cells/mL, this effect became indiscernible to controls (Figure 2C). A total quantity of 100 μg/mL FN showed potent fungicidal activity in assays with density of 10^4^ and 10^5^ cells/mL, reducing about 3 log_10_CFU at the end of assays (Figure 2A.B), but it did not reduce viable cells in assays with density of 10^6^ cells/mL (Figure 2C). Although 200 μg/mL FN demonstrated strong killing activity in all the three assays, increasing the cell density to 10^6^ cells/mL leads to more viable cells that could be detected (Figure 2), further suggesting the dependence of FN killing on initial cell density.

### 3.4 Hyphal formation

**FIGURE 3:**
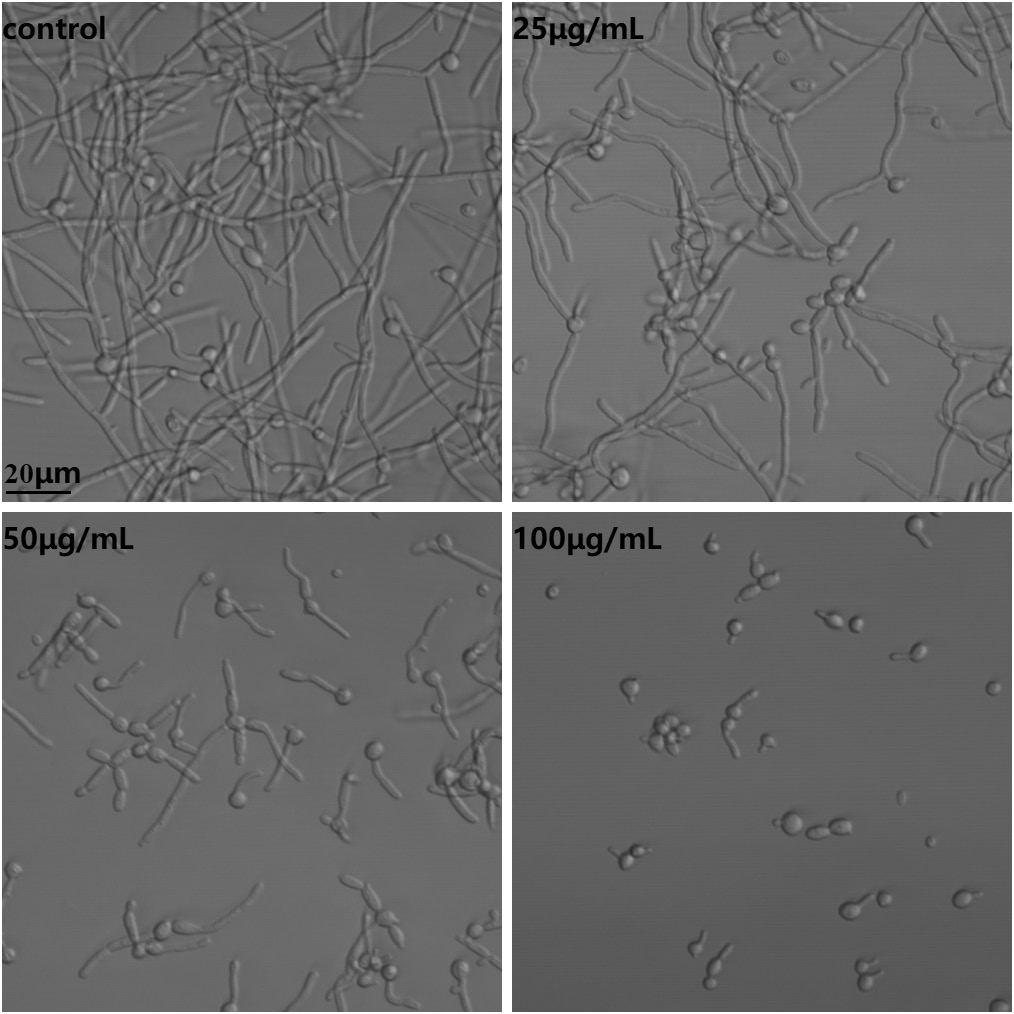
FN inhibited the hyphal growth of *Candida albicans*. *C. albicans* hyphal growth induced by RPMI‐1640 medium at 37°C for 4 h was impeded by FN.

Hyphal formation was induced in RPMI‐1640 medium at 37°C, while treatment with FN from 25 to 100 μg/mL could gradually reduce the hyphal growth. As shown in Figure 3, compared with control, treatment with 25 μg/mL FN caused more cells blocked in yeast morphology while treatment with 50 μg/mL FN not only impeded hyphal formation but also inhibited hyphal elongation. Most *Candida* cells exposed to 100 μg/mL FN were in yeast form.

### 3.5 Biofilm assay

**FIGURE 4:**
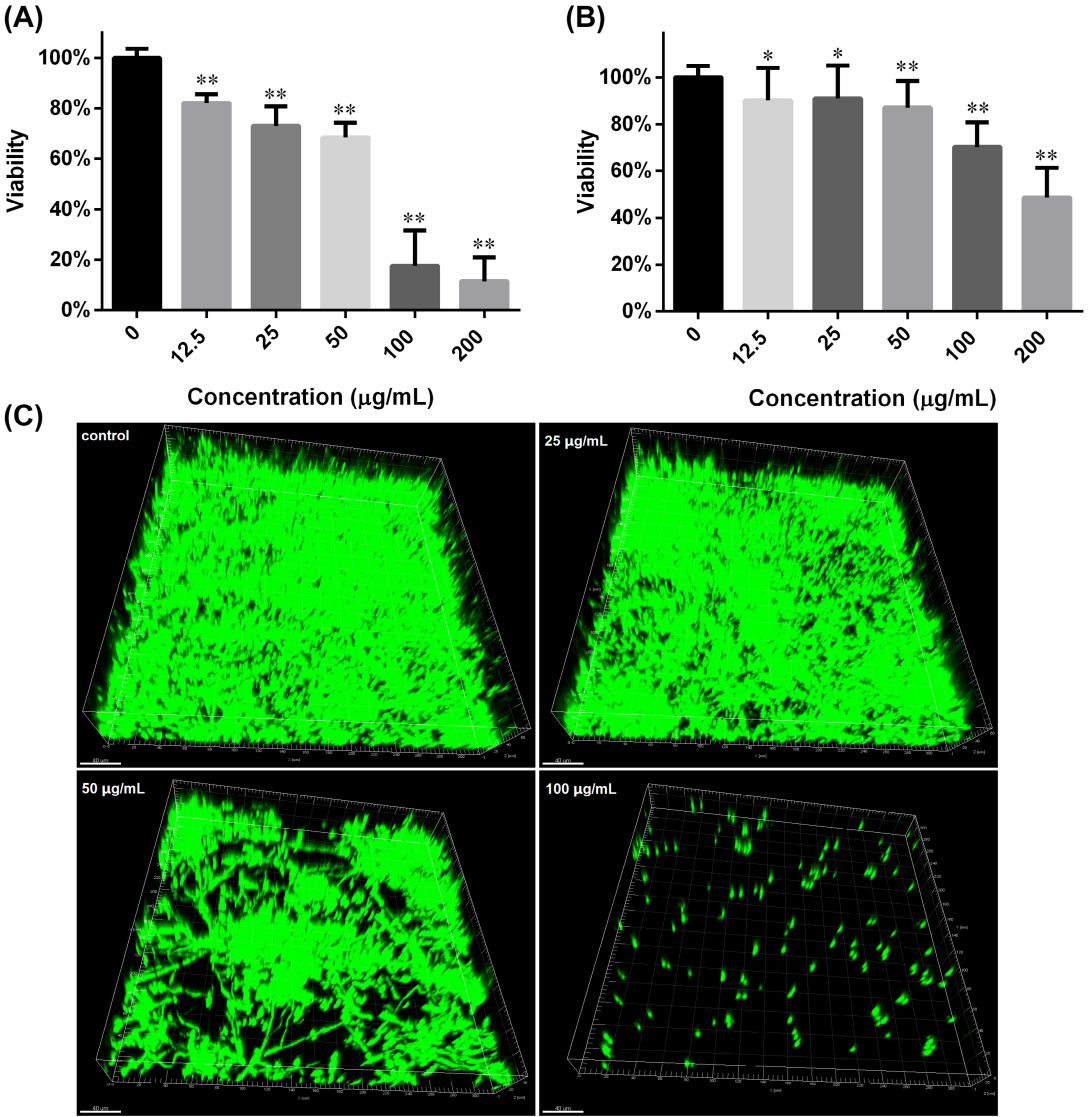
Escalating concentrations of FN inhibited both the formation and development of *Candida albicans* SC5314 biofilm. (A) The viability of *C. albicans* SC5314 biofilms formed in the presence of escalating concentrations of FN was determined through XTT reduction assay. (B) Twenty‐four hour biofilms formed in the absence of FN were exposed to FN for another 24 h. XTT assay was used to evaluate the influence of FN on the biofilm development. (C) Biofilms formed in the presence of 0, 25, 50 and 100 μg/mL FN were stained with Syto 9 and recorded by CLSM in 3D mode, followed by reconstruction using Imaris 7.2.3 software. *, *p* < 0.05, and **, *p* < 0.01.

The anti‐biofilm effects were evaluated mainly through XTT reduction assay. As revealed by Figure 4A, increasing the FN concentration from 12.5 to 200 μg/mL in the medium where *C. albicans* biofilms were formed, could decrease the biofilm viability from 82% to 11% relative to control. The half‐maximal inhibition concentration (IC_50_) fell between 50 and 100 μg/mL. As for biofilm development, the inhibition was also dose‐dependent but weaker than that of formation (Figure 4B). The IC_50_ for biofilm development was around 200 μg/mL. The results from CLSM also confirmed the inhibition of biofilm formation caused by FN (Figure 4C). Biofilms formed in presence of higher FN concentration left more void space within biofilms.

### 3.6 EPS in biofilms

**FIGURE 5:**
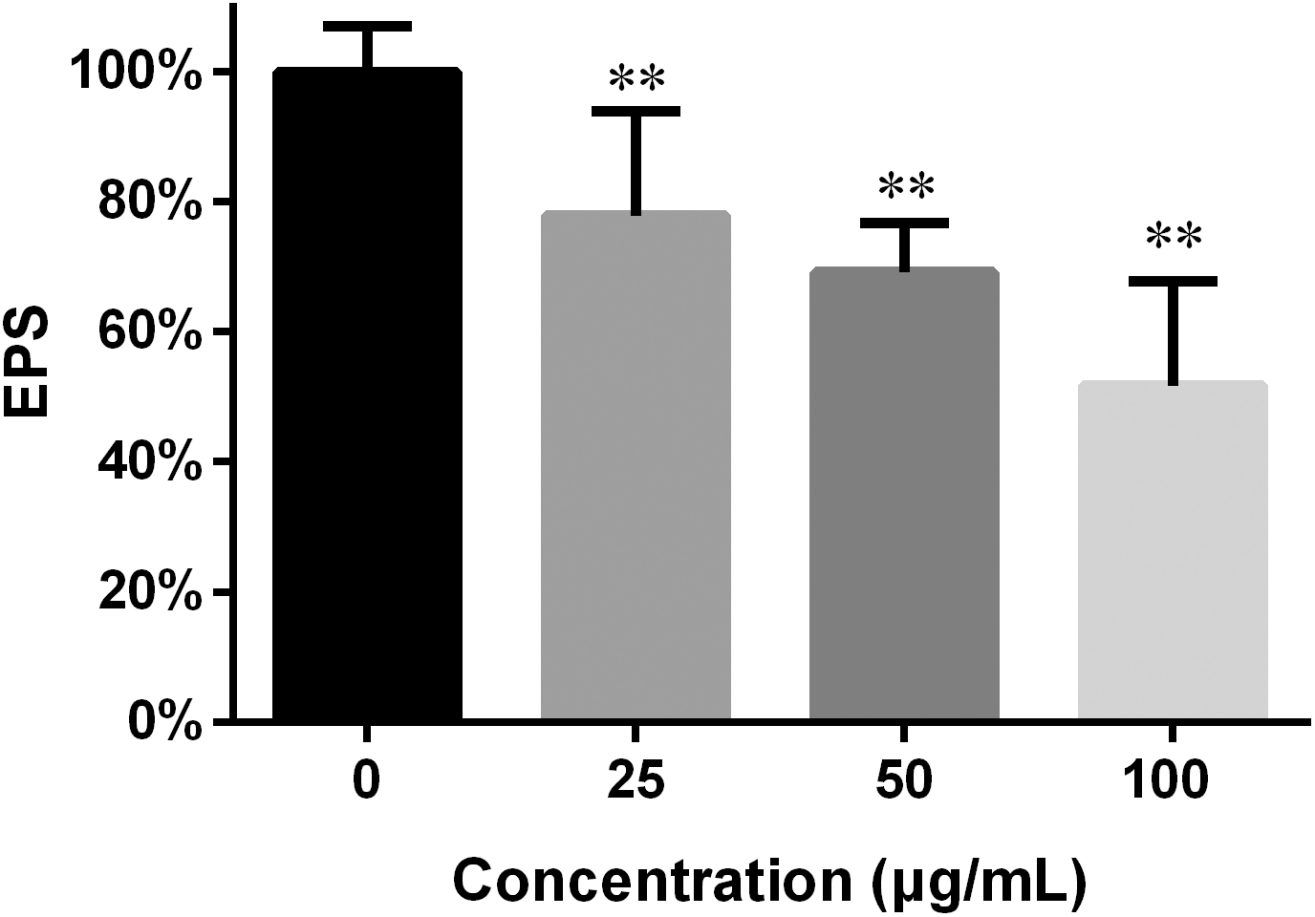
The presence of FN in preformed biofilms attenuated the EPS production. Twenty‐four hour mature biofilms treated with 0, 25, 50 and 100 μg/mL FN for another 24 h were subjected to EPS determination. **, *p* < 0.01.

Biofilm EPS often make a hurdle to access of drugs to cells within biofilms and impede their efficacy.^2^ In this study, EPS determination was performed through colorimetry.^23^ As shown in Figure 5, the EPS in the preformed biofilms was decreased and the decrease varied in response to the FN concentration: 25, 50 and 100 μg/mL caused 22%, 30% and 49% reduction in EPS respectively.

### 3.7 Cell membrane damage

**FIGURE 6:**
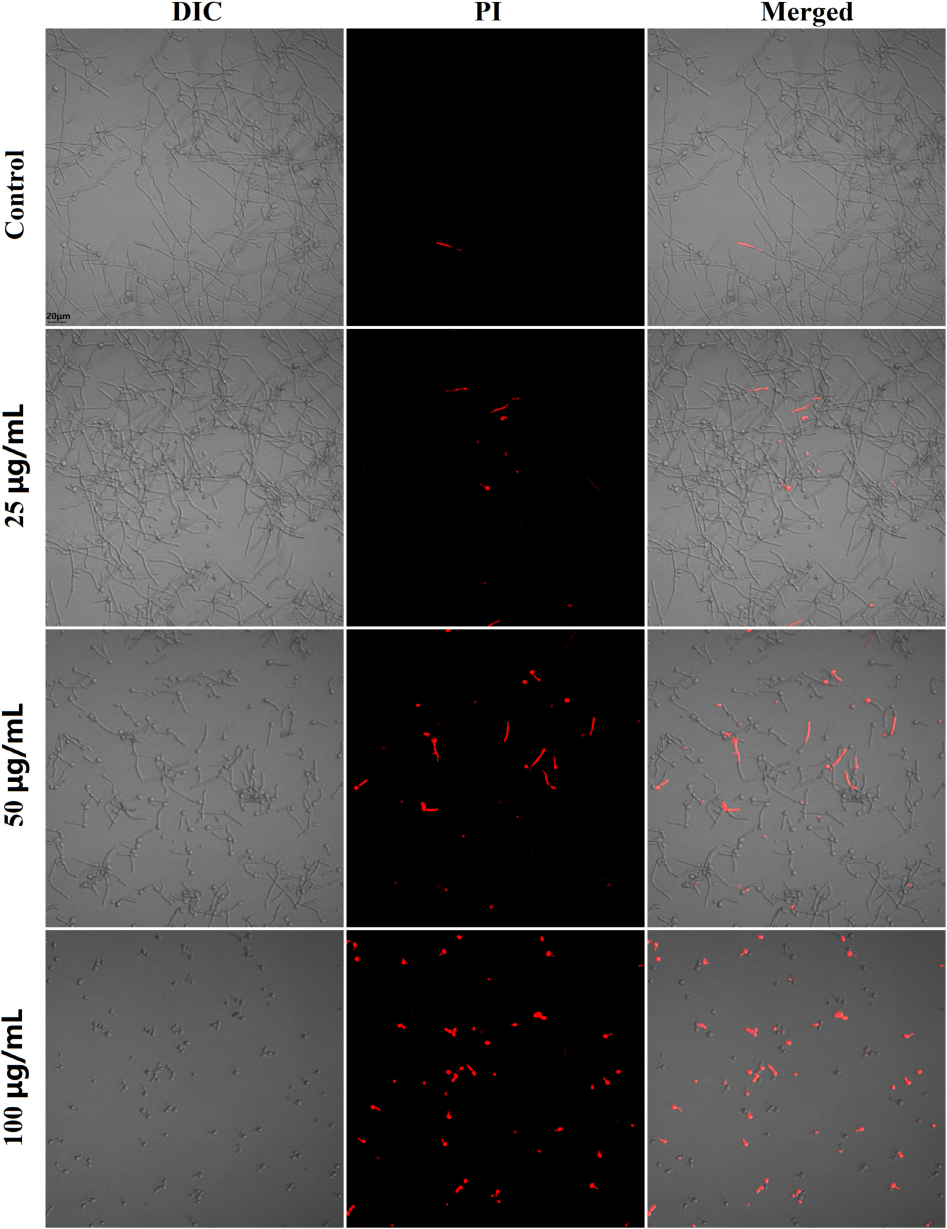
FN induced hyper‐permeability of *Candida albicans* SC5314 cell membrane. After a 4‐h co‐incubation with 0, 25, 50 and 100 μg/mL FN at 37°C, *C. albicans* cells were stained with PI for detecting cell membrane damages.

Cell membrane is important for living cells as it keeps life processes, such as metabolism, catabolism and protein synthesis, from being disturbed directly by outside. Many antimicrobials can cause damages to cell membrane, making membrane more permeable to intracellular contents. As revealed by Figure 6, the 4‐h treatment with 25, 50 and 100 μg/mL FN could gradually increase the cells that emitted red fluorescence, suggesting damages to cells membrane.

### 3.8 ROS production

**FIGURE 7:**
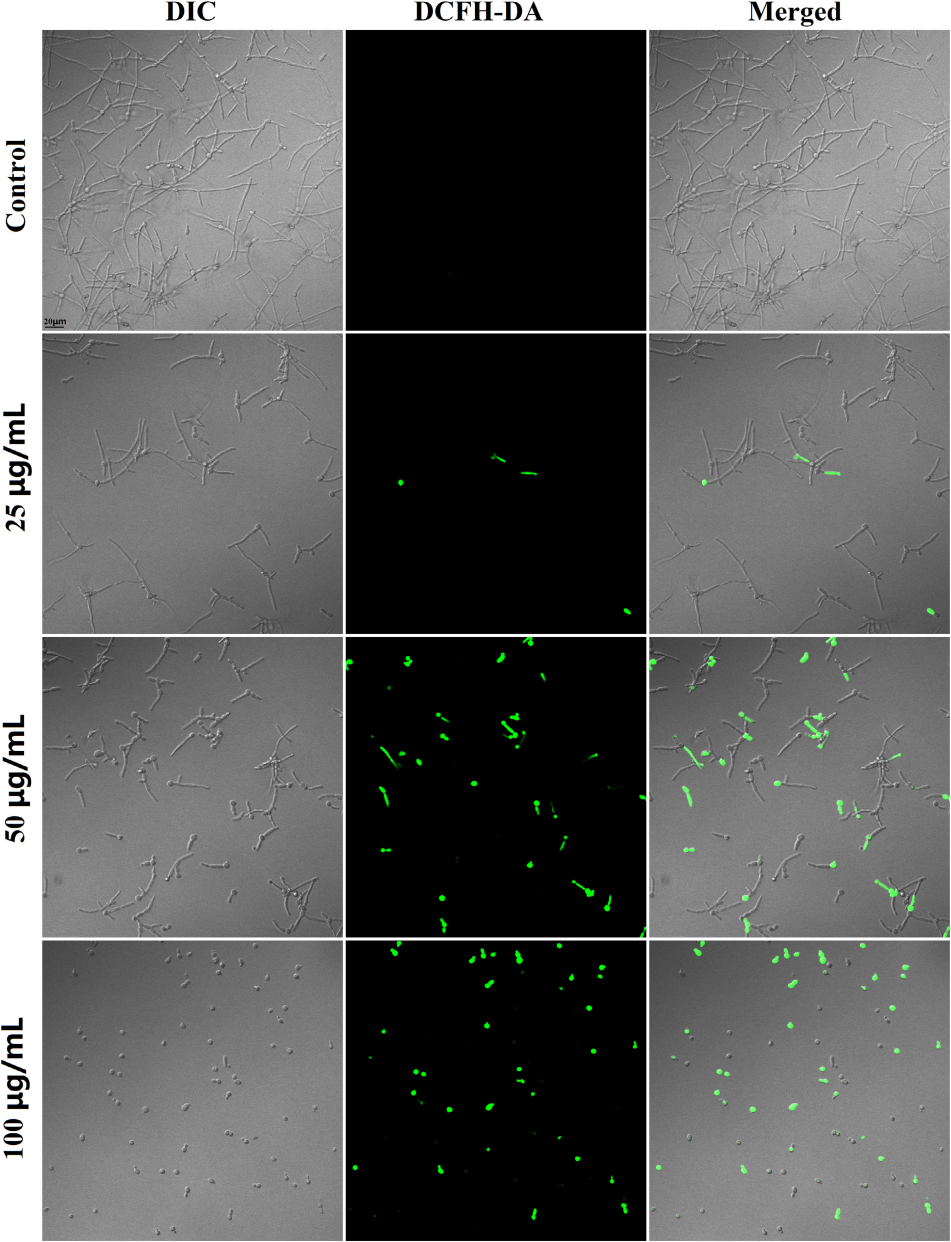
FN induced ROS overproduction in *Candida albicans* cells. DCFH‐DA was used to stain the ROS produced in *C. albicans* cells challenged with 0, 25, 50 and 100 μg/mL FN for 4 h at 37°C.

Since FN has been found to induce ROS overproduction in mammalian cancer cells,^27^ we further proceeded to explore its ROS‐inducing effects in *C. albicans* SC5314 using fluorescent dye. As shown in Figure 7, the fungal cells with green fluorescence indicating ROS production were increased as the concentration of FN was elevated.

### 3.9 NAC rescue

**FIGURE 8:**
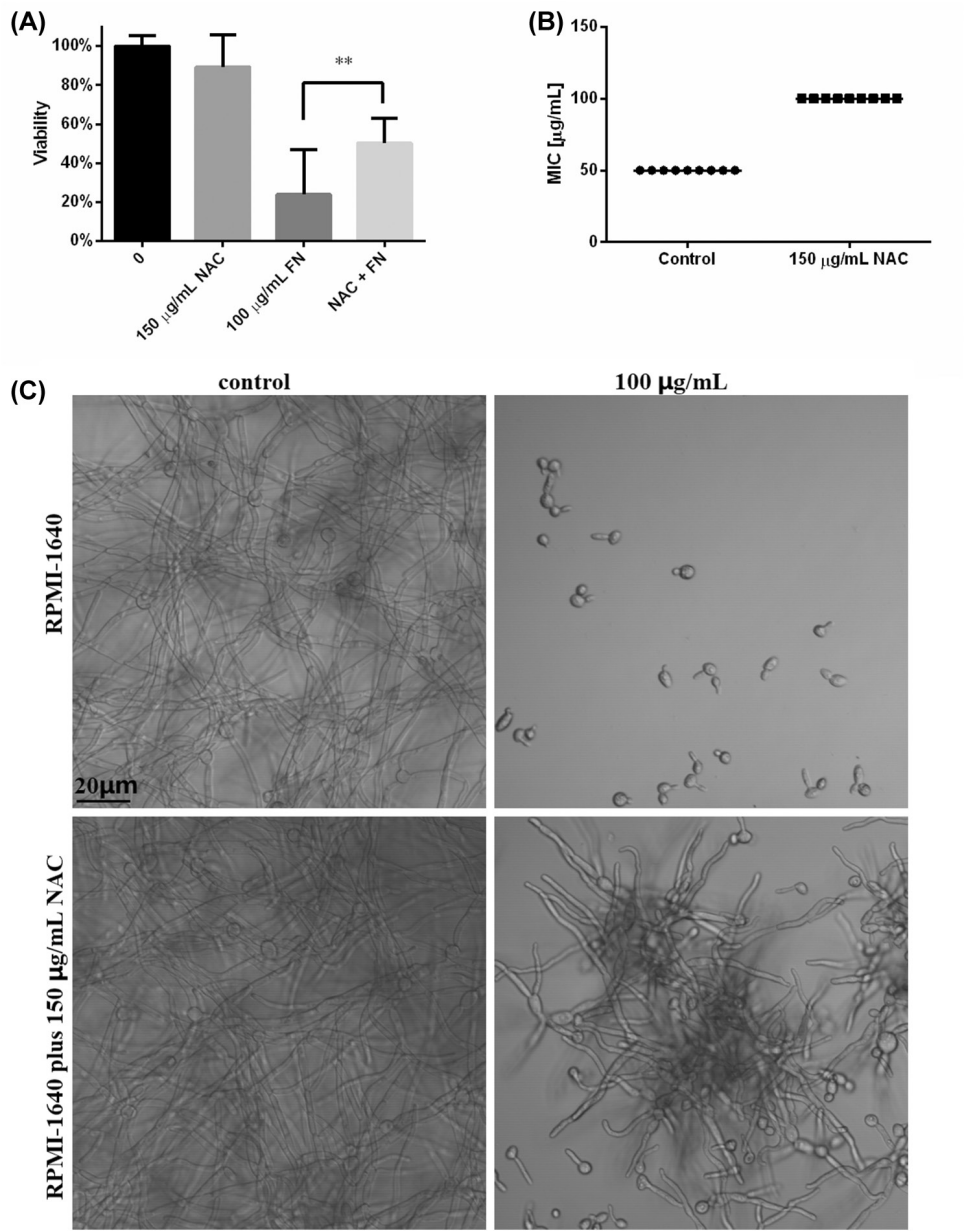
NAC rescued biofilm inhibition caused by FN treatment. (A) Addition of 150 μg/mL NAC could save part of viability of *Candida albicans* biofilms treated with 100 μg/mL FN. **, *p* < 0.01. (B) The presence of 150 μg/mL NAC could increase the MIC of FN. (C) Representative graphs of *C. albicans* biofilms formed in the absence and presence of NAC and exposed to 0 and 100 μg/mL FN.

As ROS overproduction was confirmed in FN‐treated *C. albicans* cells, we further performed rescue assay with 150 μg/mL NAC in biofilm formation protocols. The biofilms formed in the presence of NAC was lower in viability than drug‐free control, which was similar to our previous data.^26^ The presence of 100 μg/mL FN greatly impeded the biofilm formation (Figure 8A), consistent with the results of Figure 4A. As expected, the presence of NAC in the process of biofilm formation significantly saved the viability of biofilms treated with 100 μg/mL FN (Figure 8A). This saving found in viability test was also confirmed by biofilm morphology photographs, as shown in Figure 8C. In addition, the presence of 150 μg/mL NAC could also increase the MIC of FN from 50 μg/mL to 100 μg/mL (Figure 8B), further confirming the role of ROS in the anti‐*Candida* action of FN.

### 3.10 Drug combination test

Fractional inhibitory concentration index (FICI) was used to interpret the interactions between FN and antifungal drugs. As shown in Table 2, FN can synergize with all the three drugs, with FICI below 0.5.

**TABLE 2:** Interactions of FN with antifungal drugs.

Drugs	MIC of drugs		MIC of FN		FICI	Interaction
	Alone	Combined	Alone	Combined		
Amphotericin B	0.625	0.156	50	6.25	0.375	Synergistic
Caspofungin	0.625	0.156	50	3.125	0.3125	Synergistic
Fluconazole	2.5	0.3125	50	6.25	0.25	Synergistic

## 4 DISCUSSION

In spite of the increase in the infections caused by non‐albicans *Candida* species, the incidence of *C. albicans* infections is still the highest among all *Candida* species.^28^ The mortality of bloodstream infections due to *C. albicans* was reported to be as high as 40%.^2^ The treatment for *C. albicans*, and other fungal pathogens, is hard to be considered as successful, due to the lack of antifungal drugs and emergence of resistance.^2^, ^10^ The occurrence of drug‐resistant *Candida auris* further aggregates the public concern of antifungal shortage.^10^ Natural products from herbal plants have continually showed significant antifungal activities against *Candida* species, as well as other pharmacological activities.^2^, ^5^, ^29^, ^30^, ^31^ Examples include dioscin, dracorhodin perchlorate, alantolactone, carnosol and polyphyllin I, as reported previously.^7^, ^24^, ^30^, ^32^, ^33^

FN is a bis‐benzylisoquinoline moiety‐containing alkaloid, structurally similar to tetrandrine which has demonstrated antifungal activities against *C. albicans* in several studies.^16^, ^22^, ^34^ FN owns multiple bioactivities, including antitumour, anti‐inflammatory and antiviral activities,^13^, ^15^, ^18^ but its activity against human fungal pathogens remains to be explored. Given that FN and tetrandrine share similar chemical structures, and that tetrandrine showed activity against *C. albicans*,^16^, ^34^ we reasoned that FN is possible to have similar anti‐*Candida* activity. Results from CSLI‐based methods showed, for the first time, that FN has antifungal activities against all *Candida* strains (*C. albicans*, *C. krusei*, *C. glabrata*, *C. tropicalis* and *C. parapsilosis*) tested. The MIC of FN we obtained were much lower than those against plant fungal pathogens,^22^ these differences could be explained by the far evolutionary distance between them.

*C. albicans* biofilms formed on medical devices are calcitrant to azole drugs, which makes agents with anti‐biofilm activity appealing.^2^ In this study, FN not only displayed activity in planktonic growth, but also showed anti‐biofilm effects. The FN concentration required for about half maximal inhibition (IC_50_) of biofilm formation was higher than its MIC, and IC_50_ for biofilm development was even higher. This is often seen in many antifungal agents.^8^, ^26^, ^32^, ^35^ However, the ratio of IC_50_ (in biofilm formation assay) to MIC (in planktonic growth) for FN was lower than those reported for other drugs, which were over 1000 for azoles.^8^ This indicated the future anti‐biofilm potential of FN. Biofilms are formed starting from adhesion to surfaces and FN could inhibit adhesion of *C. albicans* to polystyrene surfaces. The ability to form hyphae is considered as critical for *C. albicans* pathogenicity and hyphae of *C. albicans* can reinforce the biofilm structure and confer resistance to host immune attack.^2^, ^36^ Their length often makes phagocytosis by immune cells hard and elongating may pierce membranes of macrophages. Hyphae‐associated superoxide dismutase 5 (SOD5) is useful to counter the oxidative stress (produced by immune cells or drugs). In addition, the toxin candidalysin secreted by hyphae can cause macrophage death and tissue damages.^6^, ^37^ In this scenario, the ability of FN to impede hyphal growth indicates its potential to block *C. albicans* invasion in tissues.

EPS block the access of drugs into cells within biofilms, providing another mechanism for the elevated dose needed for suppressing biofilms. It was reported FerlONP exerted EPS‐degrading effects on bacterial biofilms by in situ ROS (hydrogen peroxide) production.^38^ Therefore, it is possible that FN decrease the EPS production through similar ROS overproduction, although the reaction environments were different. It is also possible that when FN and other antifungal drugs were combined, the efficacy would be enhanced, as was the case with alantolactone and amphotericin B.^26^ Therefore, the drug combination tests were performed, and the results we obtained showed that FN, combined with amphotericin B, caspofungin or fluconazole, could produce a synergistic interaction, indicating its potential in lowering the dose of antifungals.

Many antifungal agents, such as miconazole, *θ*‐defensins, caspofungin and others, exert their effects through excessive ROS accumulation in fungal cells,^23^, ^26^, ^39^, ^40^, ^41^ and in Jurkat T cells and human multiple myeloma U266 cells, FN was reported to be able to stimulate the overproduction of intracellular ROS,^42^, ^43^ so we postulated that FN may also incur excessive ROS production in *C. albicans*. DCFH‐DA staining and NAC rescue assays confirmed the role of ROS in the antifungal and anti‐biofilm activity of FN. Although FN has been shown to have protective roles in neurodegenerative models through attenuating oxidative stress,^44^ the concentrations in that research was lower than the concentrations incurring ROS in our and other studies.^42^, ^43^ The massive production of ROS would damage biomacromolecules that build up organelles and are important for metabolism, resulting in cell death finally. In this study, FN treatment increased the permeability of *C. albicans* cell membrane, indicating its damages to cell membrane. However, whether this effect was due to ROS caused by FN or due to the interaction between FN and the phospholipids or proteins within cell membrane, remains to be explored.

Although FN was effective in this study, the concentrations used here was a little higher, compared with other antifungal agents such as dioscin and polyphyllin I, posing a need for the optimization of its chemical structure. Fortunately, there are many natural analogues of FN, such as tetrandrine and cepharanthine.^45^ In addition, lots of FN derivatives have been synthesized and the strategy for the total synthesis of bis‐benzylisoquinoline alkaloids has been mature.^22^, ^45^ All these made it plausible to explore the structure–activity of this kind of alkaloids. And another encouraging thing comes from the report that FN can attenuate the *C. albicans*‐induced acute inflammation in paws of mice.^46^ And FN also showed anti‐inflammatory activity in rat model of rheumatoid arthritis.^18^ It is conceivable that in treating inflammatory diseases associated with *C. albicans* infections, such as *C. albicans* keratitis and oral candidiasis,^47^, ^48^ FN may function through two parallel ways: suppressing *C. albicans* virulence and attenuating host inflammation. However, this requires further experimental validation.

## 5 CONCLUSION

In sum, this study showed the antifungal activity of FN against several *Candida* species, including *C. albicans*, with MIC no more than 50 μg/mL. FN also suppressed hyphal growth, adhesion, biofilm formation and development, as well as EPS production. Excessive production of ROS and cell membrane damages may underlie the antifungal activity of FN against *C. albicans*. Thus, FN may be exploited for developing alternative therapeutics for *C. albicans* infections.

## AUTHOR CONTRIBUTIONS

**Longfei Yang:** Conceptualization (equal); data curation (lead); formal analysis (equal); methodology (lead); resources (equal); software (equal); validation (equal); writing – original draft (equal); writing – review and editing (equal). **Xiaonan Wang:** Investigation (equal); project administration (equal); resources (equal); supervision (equal); writing – review and editing (equal). **Zhiming Ma:** Conceptualization (equal); formal analysis (equal); investigation (equal); resources (equal); supervision (equal); writing – review and editing (equal). **Yujie Sui:** Conceptualization (equal); data curation (equal); investigation (equal); supervision (equal); validation (equal); writing – review and editing (supporting). **Xin Liu:** Conceptualization (equal); formal analysis (equal); funding acquisition (equal); investigation (equal); resources (equal); writing – original draft (equal); writing – review and editing (equal).

## CONFLICT OF INTEREST STATEMENT

The authors have no conflicts of interest to declare.

## Data Availability

All the data supporting this study could be obtained from the corresponding author upon reasonable request.
